# Supplementary material for: Adaptability and stability analyses of plants using random regression models
Source: PLoS One. 2020 Dec 2;15(12):e0233200. doi: 10.1371/journal.pone.0233200 (PMC7710123; doi:10.1371/journal.pone.0233200)
Supplement: S1 Table — (DOCX) [file pone.0233200.s001.docx]

**S1 Table: Carioca bean cultivars, institutions of origin and year of recommendation.**

| **Cultivar** | **Institution** | **Year** | **Cultivar** | **Institution** | **Year** |
| --- | --- | --- | --- | --- | --- |
| Carioca 1030 | IAC^(1)^ | 1970 | IPR Colibri | IAPAR | 2004 |
| Carioca 80 | IAC | 1980 | IPR Saracura | IAPAR | 2004 |
| IAC Ayso | IAC | 1980 | SCS Guará | EPAGRI^(9)^ | 2004 |
| IAPAR 80 | IAPAR^(2)^ | 1980 | BRSMG Pioneiro | UFV | 2005 |
| Carioca MG | UFLA^(3)^ | 1982 | IAC Votuporanga | IAC | 2005 |
| IAPAR 16 | IAPAR | 1986 | IAC-Apuã | IAC | 2005 |
| IAC Carioca | IAC | 1987 | IAC-Ybaté | IAC | 2005 |
| Rio doce | INCAPER^(4)^ | 1987 | BRS Cometa | EMBRAPA | 2006 |
| Carioca 1070 | IAC | 1989 | IPR Eldourado | IAPAR | 2006 |
| IAPAR 31 | IAPAR | 1991 | IPR Siriri | IAPAR | 2006 |
| Aporé | EMBRAPA^(5)^ | 1992 | IAC Alvorada | IAC | 2007 |
| BR- IPA Brígida | EMBRAPA | 1992 | IPR 139 | IAPAR | 2007 |
| FT bonito | FT-Sementes^(6)^ | 1992 | IPR Tangará | IAPAR | 2008 |
| IAPAR 57 | IAPAR | 1992 | BRS Estilo | EMBRAPA | 2009 |
| IAC-Aruã | IAC | 1993 | BRS Ametista | EMBRAPA | 2011 |
| Carioca Pyatã | IAC | 1994 | BRS Notável | EMBRAPA | 2011 |
| Pérola | EMBRAPA | 1994 | IAC Formoso | IAC | 2011 |
| Rudá | EPAMIG^(7)^ | 1994 | IPR Campos Gerais | IAPAR | 2011 |
| Rudá R | UFV^(8)^ | 1995 | BRSMG Madrepérola | UFV | 2012 |
| Carioca Akytá | IAC | 1996 | IAC Imperador | IAC | 2013 |
| IAPAR 81 | IAPAR | 1997 | IAC Milênio | IAC | 2013 |
| Princesa | EMBRAPA | 1997 | IPR Andorinha | IAPAR | 2013 |
| Porto Real | FT-Sementes | 1998 | IPR Curió | IAPAR | 2013 |
| BRSMG Talismã | UFLA | 2002 | VC 17* | UFV | 2013 |
| BRS Requinte | EMBRAPA | 2003 | VC15* | UFV | 2013 |
| BRS Horizonte | EMBRAPA | 2004 | IPR Bem-te-vi | IAPAR | 2014 |
| BRS Majestoso | UFLA | 2004 | IPR Quero-Quero | IAPAR | 2014 |
| BRS Pontal | EMBRAPA | 2004 | BRS UAI | UFLA | 2015 |

^(1)^Instituto Agronômico de Campinas; ^(2)^Instituto Agronômico do Paraná; ^(3)^Universidade Federal de Lavras; ^(4)^Instituto Capixaba de Pesquisa, Assistência Técnica e Extensão Rural; ^(5)^Empresa Brasileira de Pesquisa Agropecuária; ^(6)^Empresa privada; ^(7)^Empresa de Pesquisa Agropecuária de Minas Gerais; ^(8)^Universidade Federal de Viçosa; ^(9)^Empresa de Pesquisa Agropecuária e Extensão Rural de Santa Catarina. *Elite-strains of the Programa Feijão – UFV.
