# Supplementary material for: Adaptability and stability analyses of plants using random regression models
Source: PLoS One. 2020 Dec 2;15(12):e0233200. doi: 10.1371/journal.pone.0233200 (PMC7710123; doi:10.1371/journal.pone.0233200)
Supplement: S2 Table — (DOCX) [file pone.0233200.s002.docx]

**S2 Table: Black bean cultivars, institutions of origin and year of recommendation.**

| **Cultivar** | **Institution** | **Year** | **Cultivar** | **Institution** | **Year** |
| --- | --- | --- | --- | --- | --- |
| Rico 23 | UFV^(1)^ | 1959 | IAC-Maravilha | IAC | 1993 |
| Rio Tibagi | Fepagro^(2)^ | 1971 | IAPAR 65 | IAPAR | 1993 |
| Moruna | IAC^(3)^ | 1978 | Xamego | PESAGRO | 1993 |
| Capixaba Precoce | INCAPER^(4)^ | 1980 | IAC-Una | IAC | 1994 |
| IRAÍ | IPAGRO^(5)^ | 1981 | Meia Noite | EPAMIG/UFV | 1994 |
| IAPAR Rio Negro | IAPAR^(6)^ | 1983 | IPR Uirapurú | IAPAR | 2000 |
| Milionário 1732 | UFV/EPAMIG^(7)^ | 1983 | BRS Valente | EMBRAPA | 2001 |
| BR 1- Xodó | PESAGRO^(8)^ | 1985 | IPR Graúna | IAPAR | 2002 |
| BR-2 Grande Rio | PESAGRO(^9)^ | 1985 | BRS Campeiro | EMBRAPA | 2003 |
| BR-3 Ipanema | PESAGRO | 1985 | BRS Grafite | EMBRAPA | 2003 |
| FT 120 | FT-Sementes^(10)^ | 1986 | BRS Supremo | EMBRAPA | 2004 |
| IAPAR 20 | IAPAR | 1987 | IPR Chopin | IAPAR | 2004 |
| Macanudo | EMBRAPA | 1989 | IAC Tunã | IAC | 2005 |
| Pampa | EMBRAPA | 1989 | BRS Esplendor | EMBRAPA | 2006 |
| BR Barriga Verde | EMBRAPA | 1990 | IPR Tiziu | IAPAR | 2006 |
| IAPAR 44 | IAPAR | 1990 | BRS Expedito | EMBRAPA | 2007 |
| Preto Uberabinha | IPEACO^(11)^ | 1990 | IAC- Diplomata | IAC | 2007 |
| Rico 1735 | IPEACO | 1990 | IPR Gralha | IAPAR | 2007 |
| Diamante Negro | EMGOPA^(12)^ | 1991 | BRS Agreste | EMBRAPA | 2009 |
| Macotaço | EMBRAPA | 1991 | IPR Tuiuiú | IAPAR | 2010 |
| Minuano | EMBRAPA | 1991 | BRS Esteio | EMBRAPA | 2012 |
| Varre-Sai | PESAGRO | 1991 | VP 22* | UFV | 2013 |
| BR- IPA 10 | IPA^(13)^ | 1992 | VP 33* | UFV | 2013 |
| Onix | EMBRAPA | 1992 | IPR Inhambu | IAPAR | 2015 |
| Ouro Negro | UFV/EPAMIG | 1992 |  |  |  |

^(1)^Universidade Federal de Viçosa; ^(2)^Fundação Estadual de Pesquisa Agropecuária; ^(3)^Instituto Agronômico de Campinas; ^(4)^Instituto Capixaba de Pesquisa, Assistência Técnica e Extensão Rural; ^(5)^Instituto de Pesquisas Agronômicas; ^(6)^ Instituto Agronômico do Paraná; ^(7)^ Empresa de Pesquisa Agropecuária de Minas Gerais; ^(8)^Empresa de Pesquisa Agropecuária do Estado do Rio de Janeiro; ^(9)^Empresa privada; ^(10)^Empresa Brasileira de Pesquisa Agropecuária; ^(11)^Instituto de Pesquisa e Experimentação Agropecuária do Centro-Oeste; ^(12)^Empresa Goiana de Pesquisa Agropecuária; ^(13)^Instituto Agronômico de Pernambuco. *Elite-strains of the Programa Feijão – UFV.
