# Supplementary material for: Adaptability and stability analyses of plants using random regression models
Source: PLoS One. 2020 Dec 2;15(12):e0233200. doi: 10.1371/journal.pone.0233200 (PMC7710123; doi:10.1371/journal.pone.0233200)
Supplement: S3 Table — (DOCX) [file pone.0233200.s003.docx]

**S3 Table: Description of the trials.**

| **Trial** | **Season** | **Year** | **Experimental field¹** | **Place** | **Nº of cultivars** |
| --- | --- | --- | --- | --- | --- |
| 1 | Dry | 2013 | UEPE² Coimbra | Coimbra, MG, BR | 80 |
| 2 | Dry | 2013 | Vale da Agronomia | Viçosa, MG, BR | 80 |
| 3 | Winter | 2013 | UEPE Coimbra | Coimbra, MG, BR | 80 |
| 4 | Winter | 2013 | Vale da Agronomia | Viçosa, MG, BR | 80 |
| 5 | Dry | 2015 | UEPE Coimbra | Coimbra, MG, BR | 100 |
| 6 | Winter | 2015 | UEPE Coimbra | Coimbra, MG, BR | 100 |
| 7 | Dry | 2016 | UEPE Coimbra | Coimbra, MG, BR | 100 |
| 8 | Dry | 2016 | Aeroporto | Viçosa, MG, BR | 104 |
| 9 | Dry | 2016 | UEPE Coimbra | Coimbra, MG, BR | 104 |
| 10 | Winter | 2016 | UEPE Coimbra | Coimbra, MG, BR | 104 |
| 11 | Winter | 2016 | Horta Nova | Viçosa, MG, BR | 104 |
| 12 | Dry | 2017 | Aeroporto | Viçosa, MG, BR | 105 |
| 13 | Winter | 2017 | UEPE Coimbra | Coimbra, MG, BR | 105 |

¹The experimental stations belong to the Agronomy Department of the Federal University of Viçosa (UFV); ²Education, Research and Extension Unit.
