# Supplementary material for: Adaptability and stability analyses of plants using random regression models
Source: PLoS One. 2020 Dec 2;15(12):e0233200. doi: 10.1371/journal.pone.0233200 (PMC7710123; doi:10.1371/journal.pone.0233200)
Supplement: S4 Table — (DOCX) [file pone.0233200.s004.docx]

**S4 Table: Accuracy of 105 cultivars in each trial.**

|  | **Trials** | | | | | | | | | | | | |
| --- | --- | --- | --- | --- | --- | --- | --- | --- | --- | --- | --- | --- | --- |
| **Cultivar** | **1** | **2** | **3** | **4** | **5** | **6** | **7** | **8** | **9** | **10** | **11** | **12** | **13** |
| Capixaba Precoce | 0.97 | 0.98 | 0.98 | 0.98 | 0.96 | 0.90 | 0.98 | 0.90 | 0.97 | 0.97 | 0.93 | 0.84 | 0.80 |
| Ouro Negro | 0.97 | 0.98 | 0.98 | 0.98 | 0.96 | 0.90 | 0.98 | 0.90 | 0.97 | 0.97 | 0.93 | 0.84 | 0.80 |
| Pérola | 0.97 | 0.98 | 0.98 | 0.98 | 0.96 | 0.90 | 0.98 | 0.90 | 0.97 | 0.97 | 0.93 | 0.84 | 0.80 |
| BRS Valente | 0.97 | 0.98 | 0.98 | 0.98 | 0.96 | 0.90 | 0.98 | 0.90 | 0.97 | 0.97 | 0.93 | 0.84 | 0.80 |
| BRS Campeiro | 0.97 | 0.98 | 0.98 | 0.98 | 0.96 | 0.90 | 0.98 | 0.90 | 0.97 | 0.97 | 0.93 | 0.84 | 0.80 |
| BRS Grafite | 0.97 | 0.98 | 0.98 | 0.98 | 0.96 | 0.90 | 0.98 | 0.90 | 0.97 | 0.97 | 0.93 | 0.84 | 0.80 |
| BRS Requinte | 0.97 | 0.98 | 0.98 | 0.98 | 0.96 | 0.90 | 0.98 | 0.90 | 0.97 | 0.97 | 0.93 | 0.84 | 0.80 |
| BRS Pontal | 0.97 | 0.98 | 0.98 | 0.98 | 0.96 | 0.90 | 0.98 | 0.90 | 0.97 | 0.97 | 0.93 | 0.84 | 0.80 |
| BRS Majestoso | 0.97 | 0.98 | 0.98 | 0.98 | 0.96 | 0.90 | 0.98 | 0.90 | 0.97 | 0.97 | 0.93 | 0.84 | 0.80 |
| BRS Supremo | 0.97 | 0.98 | 0.98 | 0.98 | 0.96 | 0.90 | 0.98 | 0.90 | 0.97 | 0.97 | 0.93 | 0.84 | 0.80 |
| BRSMG Pioneiro | 0.97 | 0.98 | 0.98 | 0.98 | 0.96 | 0.90 | 0.98 | 0.90 | 0.97 | 0.97 | 0.93 | 0.84 | 0.80 |
| BRS Esplendor | 0.97 | 0.98 | 0.98 | 0.98 | 0.96 | 0.90 | 0.98 | 0.90 | 0.97 | 0.97 | 0.93 | 0.84 | 0.80 |
| BRS Cometa | 0.97 | 0.98 | 0.98 | 0.98 | 0.96 | 0.90 | 0.98 | 0.90 | 0.97 | 0.97 | 0.93 | 0.84 | 0.80 |
| BRS Expedito | 0.97 | 0.98 | 0.98 | 0.98 | 0.96 | 0.90 | 0.98 | 0.90 | 0.97 | 0.97 | 0.93 | 0.84 | 0.80 |
| BRS Estilo | 0.97 | 0.98 | 0.98 | 0.98 | 0.96 | 0.90 | 0.98 | 0.90 | 0.97 | 0.97 | 0.93 | 0.84 | 0.80 |
| BRS Notável | 0.97 | 0.98 | 0.98 | 0.98 | 0.96 | 0.90 | 0.98 | 0.90 | 0.97 | 0.97 | 0.93 | 0.84 | 0.80 |
| Rio doce | 0.97 | 0.98 | 0.98 | 0.98 | 0.96 | 0.90 | 0.98 | 0.90 | 0.97 | 0.97 | 0.93 | 0.84 | 0.80 |
| Rudá | 0.97 | 0.98 | 0.98 | 0.98 | 0.96 | 0.90 | 0.98 | 0.90 | 0.97 | 0.97 | 0.93 | 0.84 | 0.80 |
| Diamante Negro | 0.97 | 0.98 | 0.98 | 0.98 | 0.96 | 0.90 | 0.98 | 0.90 | 0.97 | 0.97 | 0.93 | 0.84 | 0.80 |
| Onix | 0.97 | 0.98 | 0.98 | 0.98 | 0.96 | 0.90 | 0.98 | 0.90 | 0.97 | 0.97 | 0.93 | 0.84 | 0.80 |
| Aporé | 0.97 | 0.98 | 0.98 | 0.98 | 0.96 | 0.90 | 0.98 | 0.90 | 0.97 | 0.97 | 0.93 | 0.84 | 0.80 |
| Xamego | 0.97 | 0.98 | 0.98 | 0.98 | 0.96 | 0.90 | 0.98 | 0.90 | 0.97 | 0.97 | 0.93 | 0.84 | 0.80 |
| BR-6 Barriga verde | 0.97 | 0.98 | 0.98 | 0.98 | 0.96 | 0.90 | 0.98 | 0.90 | 0.97 | 0.97 | 0.93 | 0.84 | 0.80 |
| SCS Guará | 0.97 | 0.98 | 0.98 | 0.98 | 0.96 | 0.90 | 0.98 | 0.90 | 0.97 | 0.97 | 0.93 | 0.84 | 0.80 |
| VP 33 | 0.97 | 0.98 | 0.98 | 0.98 | 0.96 | 0.90 | 0.98 | 0.90 | 0.97 | 0.97 | 0.93 | 0.84 | 0.80 |
| VC 15 | 0.97 | 0.98 | 0.98 | 0.98 | 0.96 | 0.90 | 0.98 | 0.90 | 0.97 | 0.97 | 0.93 | 0.84 | 0.80 |
| VP 22 | 0.97 | 0.98 | 0.98 | 0.98 | 0.96 | 0.90 | 0.98 | 0.90 | 0.97 | 0.97 | 0.93 | 0.84 | 0.80 |
| Milionário 1732 | 0.97 | 0.98 | 0.98 | 0.98 | 0.96 | 0.90 | 0.98 | 0.90 | 0.97 | 0.97 | 0.93 | 0.84 | 0.80 |
| Rico 1735 | 0.97 | 0.98 | 0.98 | 0.98 | 0.96 | 0.90 | 0.98 | 0.90 | 0.97 | 0.97 | 0.93 | 0.84 | 0.80 |
| FT 120 | 0.97 | 0.98 | 0.98 | 0.98 | 0.96 | 0.90 | 0.98 | 0.90 | 0.97 | 0.97 | 0.93 | 0.84 | 0.80 |
| FT bonito | 0.97 | 0.98 | 0.98 | 0.98 | 0.96 | 0.90 | 0.98 | 0.90 | 0.97 | 0.97 | 0.93 | 0.84 | 0.80 |
| Carioca 1070 | 0.97 | 0.98 | 0.98 | 0.98 | 0.96 | 0.90 | 0.98 | 0.90 | 0.97 | 0.97 | 0.93 | 0.84 | 0.80 |
| Carioca 1030 | 0.97 | 0.98 | 0.98 | 0.98 | 0.96 | 0.90 | 0.98 | 0.90 | 0.97 | 0.97 | 0.93 | 0.84 | 0.80 |
| Moruna | 0.97 | 0.98 | 0.98 | 0.98 | 0.96 | 0.90 | 0.98 | 0.90 | 0.97 | 0.97 | 0.93 | 0.84 | 0.80 |
| Carioca 80 | 0.97 | 0.98 | 0.98 | 0.98 | 0.96 | 0.90 | 0.98 | 0.90 | 0.97 | 0.97 | 0.93 | 0.84 | 0.80 |
| IAC Carioca | 0.97 | 0.98 | 0.98 | 0.98 | 0.96 | 0.90 | 0.98 | 0.90 | 0.97 | 0.97 | 0.93 | 0.84 | 0.80 |
| IAC-Una | 0.97 | 0.98 | 0.98 | 0.98 | 0.96 | 0.90 | 0.98 | 0.90 | 0.97 | 0.97 | 0.93 | 0.84 | 0.80 |
| Carioca Pyatã | 0.97 | 0.98 | 0.98 | 0.98 | 0.96 | 0.90 | 0.98 | 0.90 | 0.97 | 0.97 | 0.93 | 0.84 | 0.80 |
| Carioca Akytá | 0.97 | 0.98 | 0.98 | 0.98 | 0.96 | 0.90 | 0.98 | 0.90 | 0.97 | 0.97 | 0.93 | 0.84 | 0.80 |
| IAC Votuporanga | 0.97 | 0.98 | 0.98 | 0.98 | 0.96 | 0.90 | 0.98 | 0.90 | 0.97 | 0.97 | 0.93 | 0.84 | 0.80 |
| IAC-Ybaté | 0.97 | 0.98 | 0.98 | 0.98 | 0.96 | 0.89 | 0.98 | 0.90 | 0.97 | 0.97 | 0.93 | 0.79 | 0.80 |
| IAC-Apuã | 0.97 | 0.98 | 0.98 | 0.98 | 0.96 | 0.90 | 0.98 | 0.90 | 0.97 | 0.97 | 0.93 | 0.84 | 0.80 |
| IAC Alvorada | 0.97 | 0.98 | 0.98 | 0.98 | 0.96 | 0.90 | 0.98 | 0.90 | 0.97 | 0.97 | 0.93 | 0.84 | 0.80 |
| IAC Formoso | 0.97 | 0.98 | 0.98 | 0.98 | 0.96 | 0.90 | 0.98 | 0.90 | 0.97 | 0.97 | 0.93 | 0.84 | 0.80 |
| Rio Tibagi | 0.97 | 0.98 | 0.98 | 0.98 | 0.96 | 0.90 | 0.98 | 0.90 | 0.97 | 0.97 | 0.93 | 0.84 | 0.80 |
| IAPAR 8-Rio Negro | 0.97 | 0.98 | 0.98 | 0.98 | 0.96 | 0.90 | 0.98 | 0.90 | 0.97 | 0.97 | 0.93 | 0.84 | 0.80 |
| IAPAR 16 | 0.97 | 0.98 | 0.98 | 0.98 | 0.96 | 0.90 | 0.98 | 0.90 | 0.97 | 0.97 | 0.93 | 0.84 | 0.80 |
| IAPAR 20 | 0.97 | 0.98 | 0.98 | 0.98 | 0.96 | 0.90 | 0.98 | 0.90 | 0.97 | 0.97 | 0.93 | 0.84 | 0.80 |
| IAPAR 44 | 0.97 | 0.98 | 0.98 | 0.98 | 0.96 | 0.90 | 0.98 | 0.90 | 0.97 | 0.97 | 0.93 | 0.84 | 0.80 |
| IAPAR 31 | 0.97 | 0.98 | 0.98 | 0.98 | 0.96 | 0.90 | 0.98 | 0.90 | 0.97 | 0.97 | 0.93 | 0.84 | 0.80 |
| IAPAR 57 | 0.97 | 0.98 | 0.98 | 0.98 | 0.96 | 0.90 | 0.98 | 0.90 | 0.97 | 0.97 | 0.93 | 0.84 | 0.80 |
| IAPAR 65 | 0.97 | 0.98 | 0.98 | 0.98 | 0.96 | 0.89 | 0.98 | 0.90 | 0.97 | 0.97 | 0.93 | 0.84 | 0.79 |
| IPR Tangará | 0.97 | 0.98 | 0.98 | 0.98 | 0.96 | 0.90 | 0.98 | 0.90 | 0.97 | 0.97 | 0.93 | 0.84 | 0.80 |
| IPR Tuiuiú | 0.97 | 0.98 | 0.98 | 0.98 | 0.96 | 0.90 | 0.98 | 0.90 | 0.97 | 0.97 | 0.93 | 0.84 | 0.80 |
| BR IPA 10 | 0.97 | 0.98 | 0.98 | 0.98 | 0.96 | 0.90 | 0.98 | 0.90 | 0.97 | 0.97 | 0.93 | 0.84 | 0.80 |
| BR IPA Brígida | 0.97 | 0.98 | 0.98 | 0.98 | 0.95 | 0.89 | 0.98 | 0.89 | 0.97 | 0.97 | 0.93 | 0.84 | 0.79 |
| IRAÍ | 0.97 | 0.98 | 0.98 | 0.98 | 0.96 | 0.90 | 0.98 | 0.90 | 0.97 | 0.97 | 0.93 | 0.84 | 0.80 |
| IPAGRO Macanudo | 0.97 | 0.98 | 0.98 | 0.98 | 0.96 | 0.90 | 0.98 | 0.90 | 0.97 | 0.97 | 0.93 | 0.84 | 0.80 |
| Preto Uberabinha | 0.97 | 0.98 | 0.98 | 0.98 | 0.96 | 0.89 | 0.98 | 0.90 | 0.97 | 0.97 | 0.93 | 0.84 | 0.79 |
| BR-2 Grande Rio | 0.97 | 0.98 | 0.98 | 0.98 | 0.96 | 0.90 | 0.98 | 0.90 | 0.97 | 0.97 | 0.93 | 0.84 | 0.80 |
| BR-3 Ipanema | 0.97 | 0.98 | 0.98 | 0.98 | 0.96 | 0.90 | 0.98 | 0.90 | 0.97 | 0.97 | 0.93 | 0.84 | 0.80 |
| BR-1 Xodó | 0.97 | 0.98 | 0.98 | 0.98 | 0.96 | 0.90 | 0.98 | 0.90 | 0.97 | 0.97 | 0.93 | 0.84 | 0.80 |
| Varre-Sai | 0.97 | 0.98 | 0.98 | 0.98 | 0.96 | 0.90 | 0.98 | 0.90 | 0.97 | 0.97 | 0.93 | 0.84 | 0.80 |
| BRSMG Madrepérola | 0.97 | 0.98 | 0.98 | 0.98 | 0.96 | 0.90 | 0.98 | 0.90 | 0.97 | 0.97 | 0.93 | 0.84 | 0.80 |
| BRSMG Talismã | 0.97 | 0.98 | 0.98 | 0.98 | 0.96 | 0.90 | 0.98 | 0.90 | 0.97 | 0.97 | 0.93 | 0.84 | 0.80 |
| Rico 23 | 0.97 | 0.98 | 0.98 | 0.98 | 0.96 | 0.90 | 0.98 | 0.90 | 0.97 | 0.97 | 0.93 | 0.84 | 0.80 |
| IPR 139 | 0.97 | 0.98 | 0.98 | 0.98 | 0.96 | 0.90 | 0.98 | 0.90 | 0.97 | 0.97 | 0.93 | 0.84 | 0.80 |
| IPR Uirapurú | 0.97 | 0.98 | 0.98 | 0.98 | 0.96 | 0.90 | 0.98 | 0.90 | 0.97 | 0.97 | 0.93 | 0.84 | 0.80 |
| IPR Gralha | 0.97 | 0.98 | 0.98 | 0.98 | 0.96 | 0.90 | 0.98 | 0.90 | 0.97 | 0.97 | 0.93 | 0.84 | 0.80 |
| IPR Eldourado | 0.97 | 0.98 | 0.98 | 0.98 | 0.96 | 0.90 | 0.98 | 0.90 | 0.97 | 0.97 | 0.93 | 0.84 | 0.80 |
| IPR Graúna | 0.97 | 0.98 | 0.98 | 0.98 | 0.96 | 0.89 | 0.98 | 0.90 | 0.97 | 0.97 | 0.93 | 0.79 | 0.80 |
| IPR Tiziu | 0.97 | 0.98 | 0.98 | 0.98 | 0.96 | 0.90 | 0.98 | 0.90 | 0.97 | 0.97 | 0.93 | 0.84 | 0.80 |
| IPR Campos Gerais | 0.97 | 0.98 | 0.98 | 0.98 | 0.96 | 0.90 | 0.98 | 0.90 | 0.97 | 0.97 | 0.93 | 0.84 | 0.80 |
| IPR Saracura | 0.97 | 0.98 | 0.98 | 0.98 | 0.96 | 0.90 | 0.98 | 0.90 | 0.97 | 0.97 | 0.93 | 0.84 | 0.80 |
| IAPAR 81 | 0.97 | 0.98 | 0.98 | 0.98 | 0.96 | 0.90 | 0.98 | 0.90 | 0.97 | 0.97 | 0.93 | 0.84 | 0.80 |
| Pampa | 0.97 | 0.98 | 0.98 | 0.98 | 0.96 | 0.90 | 0.98 | 0.90 | 0.97 | 0.97 | 0.93 | 0.84 | 0.80 |
| IAC Tunã | 0.97 | 0.98 | 0.98 | 0.98 | 0.96 | 0.90 | 0.98 | 0.90 | 0.97 | 0.97 | 0.93 | 0.84 | 0.80 |
| IPR Andorinha | 0.97 | 0.98 | 0.98 | 0.98 | 0.96 | 0.90 | 0.98 | 0.90 | 0.97 | 0.97 | 0.93 | 0.84 | 0.80 |
| IPR Colibri | 0.97 | 0.98 | 0.98 | 0.98 | 0.96 | 0.90 | 0.98 | 0.90 | 0.97 | 0.97 | 0.93 | 0.84 | 0.80 |
| IAC Imperador | 0.97 | 0.98 | 0.98 | 0.98 | 0.96 | 0.90 | 0.98 | 0.90 | 0.97 | 0.97 | 0.93 | 0.84 | 0.80 |
| BRS Esteio | 0.89 | 0.91 | 0.90 | 0.90 | 0.91 | 0.88 | 0.90 | 0.88 | 0.89 | 0.91 | 0.88 | 0.83 | 0.80 |
| Meia Noite | 0.89 | 0.91 | 0.90 | 0.90 | 0.91 | 0.88 | 0.90 | 0.88 | 0.89 | 0.91 | 0.88 | 0.83 | 0.80 |
| Porto Real | 0.89 | 0.91 | 0.90 | 0.90 | 0.91 | 0.88 | 0.90 | 0.88 | 0.89 | 0.91 | 0.88 | 0.83 | 0.80 |
| Minuano | 0.89 | 0.91 | 0.90 | 0.90 | 0.91 | 0.88 | 0.90 | 0.88 | 0.89 | 0.91 | 0.88 | 0.83 | 0.80 |
| IAC-Aruã | 0.89 | 0.91 | 0.90 | 0.90 | 0.91 | 0.88 | 0.90 | 0.88 | 0.89 | 0.91 | 0.88 | 0.83 | 0.80 |
| BRS Agreste | 0.89 | 0.91 | 0.90 | 0.90 | 0.91 | 0.88 | 0.90 | 0.88 | 0.89 | 0.91 | 0.88 | 0.83 | 0.80 |
| IAC Ayso | 0.89 | 0.91 | 0.90 | 0.90 | 0.91 | 0.88 | 0.90 | 0.88 | 0.89 | 0.91 | 0.88 | 0.83 | 0.80 |
| Macotaço | 0.89 | 0.91 | 0.90 | 0.90 | 0.91 | 0.88 | 0.90 | 0.88 | 0.89 | 0.91 | 0.88 | 0.83 | 0.80 |
| Rudá R | 0.89 | 0.91 | 0.90 | 0.90 | 0.91 | 0.88 | 0.90 | 0.88 | 0.89 | 0.91 | 0.88 | 0.83 | 0.80 |
| IAC- Diplomata | 0.89 | 0.91 | 0.90 | 0.90 | 0.91 | 0.88 | 0.90 | 0.88 | 0.89 | 0.91 | 0.88 | 0.83 | 0.80 |
| BRS Horizonte | 0.89 | 0.91 | 0.90 | 0.90 | 0.91 | 0.88 | 0.90 | 0.88 | 0.89 | 0.91 | 0.88 | 0.83 | 0.80 |
| IAC-Maravilha | 0.89 | 0.91 | 0.90 | 0.90 | 0.91 | 0.88 | 0.90 | 0.88 | 0.89 | 0.91 | 0.88 | 0.83 | 0.80 |
| BRS Ametista | 0.89 | 0.91 | 0.90 | 0.90 | 0.91 | 0.88 | 0.90 | 0.88 | 0.89 | 0.91 | 0.88 | 0.83 | 0.80 |
| IAPAR 80 | 0.89 | 0.91 | 0.90 | 0.90 | 0.91 | 0.88 | 0.90 | 0.88 | 0.89 | 0.91 | 0.88 | 0.83 | 0.80 |
| Carioca MG | 0.88 | 0.90 | 0.89 | 0.89 | 0.90 | 0.87 | 0.88 | 0.87 | 0.88 | 0.90 | 0.87 | 0.83 | 0.80 |
| Princesa | 0.89 | 0.91 | 0.90 | 0.90 | 0.91 | 0.88 | 0.90 | 0.88 | 0.89 | 0.91 | 0.88 | 0.83 | 0.80 |
| IPR Siriri | 0.89 | 0.91 | 0.90 | 0.90 | 0.91 | 0.88 | 0.90 | 0.88 | 0.89 | 0.91 | 0.88 | 0.83 | 0.80 |
| IPR Chopim | 0.89 | 0.91 | 0.90 | 0.90 | 0.91 | 0.88 | 0.90 | 0.88 | 0.89 | 0.91 | 0.88 | 0.83 | 0.80 |
| BRS Uai | 0.89 | 0.91 | 0.90 | 0.90 | 0.91 | 0.88 | 0.90 | 0.88 | 0.89 | 0.91 | 0.88 | 0.83 | 0.80 |
| VC 17 | 0.89 | 0.91 | 0.90 | 0.90 | 0.91 | 0.88 | 0.90 | 0.88 | 0.89 | 0.91 | 0.88 | 0.83 | 0.80 |
| IPR Quero-Quero | 0.83 | 0.85 | 0.84 | 0.84 | 0.86 | 0.84 | 0.83 | 0.84 | 0.84 | 0.85 | 0.83 | 0.83 | 0.78 |
| IPR Inhambu | 0.83 | 0.85 | 0.84 | 0.84 | 0.86 | 0.84 | 0.83 | 0.84 | 0.84 | 0.85 | 0.83 | 0.83 | 0.78 |
| IPR Curió | 0.83 | 0.85 | 0.84 | 0.84 | 0.86 | 0.84 | 0.83 | 0.84 | 0.84 | 0.85 | 0.83 | 0.83 | 0.78 |
| IPR Bem-te-vi | 0.83 | 0.85 | 0.84 | 0.84 | 0.86 | 0.84 | 0.83 | 0.84 | 0.84 | 0.85 | 0.83 | 0.83 | 0.78 |
| IAC Milênio | 0.24 | 0.32 | 0.24 | 0.23 | 0.49 | 0.67 | 0.21 | 0.64 | 0.25 | 0.38 | 0.45 | 0.83 | 0.74 |

*Underlined values indicate the accuracy of cultivars in trials where they not were evaluated.
