# Supplementary material for: Adaptability and stability analyses of plants using random regression models
Source: PLoS One. 2020 Dec 2;15(12):e0233200. doi: 10.1371/journal.pone.0233200 (PMC7710123; doi:10.1371/journal.pone.0233200)
Supplement: S5 Table — (DOCX) [file pone.0233200.s005.docx]

**S5 Table: Recommendation probability values for each ideotype.**

| **Cultivars** | **Grain** | **General** | **Desfavorable** | **Favorable** |
| --- | --- | --- | --- | --- |
| Capixaba Precoce | Black | 0.005 | 0.005 | 0.005 |
| Ouro Negro | Black | 0.006 | 0.006 | 0.006 |
| Pérola | Carioca | 0.009 | 0.009 | 0.008 |
| BRS Valente | Black | 0.008 | 0.008 | 0.008 |
| BRS Campeiro | Black | 0.011 | 0.011 | 0.011 |
| BRS Grafite | Black | 0.009 | 0.010 | 0.008 |
| BRS Requinte | Carioca | 0.009 | 0.009 | 0.009 |
| BRS Pontal | Carioca | 0.013 | 0.013 | 0.012 |
| BRS Majestoso | Carioca | 0.011 | 0.011 | 0.010 |
| BRS Supremo | Black | 0.009 | 0.010 | 0.008 |
| BRSMG Pioneiro | Carioca | 0.010 | 0.010 | 0.009 |
| BRS Esplendor | Black | 0.012 | 0.012 | 0.011 |
| BRS Cometa | Carioca | 0.008 | 0.008 | 0.007 |
| BRS Expedito | Black | 0.013 | 0.013 | 0.012 |
| BRS Estilo | Carioca | 0.027 | 0.024 | 0.027 |
| BRS Notável | Carioca | 0.016 | 0.016 | 0.015 |
| Rio doce | Carioca | 0.006 | 0.006 | 0.006 |
| Rudá | Carioca | 0.006 | 0.006 | 0.005 |
| Diamante Negro | Black | 0.010 | 0.009 | 0.009 |
| Onix | Black | 0.006 | 0.006 | 0.006 |
| Aporé | Carioca | 0.007 | 0.007 | 0.007 |
| Xamego | Black | 0.006 | 0.007 | 0.006 |
| BR-6 Barriga verde | Black | 0.006 | 0.006 | 0.006 |
| SCS Guará | Carioca | 0.008 | 0.008 | 0.007 |
| VP 33 | Black | 0.012 | 0.013 | 0.011 |
| VC 15 | Carioca | 0.027 | 0.023 | 0.029 |
| VP 22 | Black | 0.023 | 0.021 | 0.023 |
| Milionário 1732 | Black | 0.006 | 0.007 | 0.006 |
| Rico 1735 | Black | 0.006 | 0.006 | 0.005 |
| FT 120 | Black | 0.006 | 0.006 | 0.005 |
| FT bonito | Carioca | 0.006 | 0.006 | 0.005 |
| Carioca 1070 | Carioca | 0.005 | 0.005 | 0.004 |
| Carioca 1030 | Carioca | 0.006 | 0.006 | 0.005 |
| Moruna | Black | 0.005 | 0.006 | 0.005 |
| Carioca 80 | Carioca | 0.007 | 0.007 | 0.006 |
| IAC Carioca | Carioca | 0.006 | 0.006 | 0.006 |
| IAC-Una | Black | 0.009 | 0.009 | 0.009 |
| IAC Carioca Pyatã | Carioca | 0.007 | 0.007 | 0.006 |
| IAC Carioca Akytá | Carioca | 0.008 | 0.008 | 0.008 |
| IAC Votuporanga | Carioca | 0.011 | 0.011 | 0.010 |
| **Cultivars** | **Grain** | **General** | **Desfavorable** | **Favorable** |
| IAC-Ybaté | Carioca | 0.011 | 0.011 | 0.010 |
| IAC-Apuã | Carioca | 0.012 | 0.013 | 0.011 |
| IAC Alvorada | Carioca | 0.012 | 0.013 | 0.011 |
| IAC Formoso | Carioca | 0.032 | 0.021 | 0.096 |
| Rio Tibagi | Black | 0.007 | 0.007 | 0.006 |
| IAPAR Rio Negro | Black | 0.005 | 0.006 | 0.005 |
| IAPAR 16 | Carioca | 0.005 | 0.005 | 0.004 |
| IAPAR 20 | Black | 0.006 | 0.007 | 0.006 |
| IAPAR 44 | Black | 0.007 | 0.007 | 0.006 |
| IAPAR 31 | Carioca | 0.005 | 0.005 | 0.005 |
| IAPAR 57 | Carioca | 0.005 | 0.005 | 0.004 |
| IAPAR 65 | Black | 0.007 | 0.007 | 0.006 |
| IPR Tangará | Carioca | 0.016 | 0.017 | 0.014 |
| IPR Tuiuiú | Black | 0.026 | 0.035 | 0.021 |
| BR IPA 10 | Black | 0.006 | 0.007 | 0.006 |
| BR IPA 11-Brígida | Carioca | 0.006 | 0.006 | 0.006 |
| IRAÍ | Black | 0.006 | 0.007 | 0.006 |
| BR-IPAGRO Macanudo | Black | 0.007 | 0.007 | 0.006 |
| Preto Uberabinha | Black | 0.005 | 0.005 | 0.005 |
| BR-2 Grande Rio | Black | 0.006 | 0.006 | 0.005 |
| BR-3 Ipanema | Black | 0.005 | 0.006 | 0.005 |
| BR-1 Xodó | Black | 0.007 | 0.007 | 0.006 |
| Varre-Sai | Black | 0.006 | 0.007 | 0.006 |
| BRSMG Madrepérola | Carioca | 0.015 | 0.015 | 0.015 |
| BRSMG Talismã | Carioca | 0.009 | 0.009 | 0.008 |
| Rico 23 | Black | 0.006 | 0.006 | 0.005 |
| IPR 139 | Carioca | 0.016 | 0.013 | 0.017 |
| IPR Uirapurú | Black | 0.011 | 0.011 | 0.010 |
| IPR Gralha | Black | 0.010 | 0.011 | 0.009 |
| IPR Eldourado | Carioca | 0.008 | 0.008 | 0.008 |
| IPR Graúna | Black | 0.009 | 0.009 | 0.008 |
| IPR Tiziu | Black | 0.017 | 0.018 | 0.015 |
| IPR Campos Gerais | Carioca | 0.061 | 0.055 | 0.061 |
| IPR Saracura | Carioca | 0.009 | 0.009 | 0.008 |
| IAPAR 81 | Carioca | 0.009 | 0.008 | 0.008 |
| Pampa | Black | 0.007 | 0.007 | 0.006 |
| IAC Tunã | Black | 0.014 | 0.013 | 0.014 |
| IPR Andorinha | Carioca | 0.017 | 0.018 | 0.016 |
| IPR Colibri | Carioca | 0.007 | 0.008 | 0.007 |
| IAC Imperador | Carioca | 0.018 | 0.020 | 0.016 |
| BRS Esteio | Black | 0.010 | 0.011 | 0.009 |
| Meia Noite | Black | 0.007 | 0.007 | 0.006 |
| **Cultivars** | **Grain** | **General** | **Desfavorable** | **Favorable** |
| Porto Real | Carioca | 0.006 | 0.007 | 0.006 |
| Minuano | Black | 0.009 | 0.009 | 0.008 |
| IAC-Aruã | Carioca | 0.005 | 0.006 | 0.005 |
| BRS Agreste | Black | 0.017 | 0.015 | 0.017 |
| IAC Ayso | Carioca | 0.009 | 0.009 | 0.009 |
| Macotaço | Black | 0.007 | 0.007 | 0.006 |
| Rudá R | Carioca | 0.010 | 0.010 | 0.009 |
| IAC- Diplomata | Black | 0.008 | 0.008 | 0.007 |
| BRS Horizonte | Carioca | 0.005 | 0.005 | 0.004 |
| IAC-Maravilha | Black | 0.005 | 0.005 | 0.004 |
| BRS Ametista | Carioca | 0.008 | 0.008 | 0.008 |
| IAPAR 80 | Carioca | 0.006 | 0.006 | 0.005 |
| Carioca MG | Carioca | 0.007 | 0.008 | 0.007 |
| Princesa | Carioca | 0.006 | 0.006 | 0.006 |
| IPR Siriri | Carioca | 0.009 | 0.008 | 0.009 |
| IPR Chopim | Black | 0.013 | 0.014 | 0.012 |
| BRS Uai | Carioca | 0.009 | 0.009 | 0.008 |
| VC 17 | Carioca | 0.010 | 0.010 | 0.008 |
