# Supplementary material for: Adaptability and stability analyses of plants using random regression models
Source: PLoS One. 2020 Dec 2;15(12):e0233200. doi: 10.1371/journal.pone.0233200 (PMC7710123; doi:10.1371/journal.pone.0233200)
Supplement: S1 Code — (DOCX) [file pone.0233200.s006.docx]

**S1 Code: script for analyses.**

We used and recommend the use of ASReml-R and R Stats packages.

packagesrm(list=ls())

LPOLY = function(no) {

if(no > 9 ) no = 9

nom = no - 1

phi = matrix(data=c(0),nrow=9,ncol=9)

phi[1,1]=1

phi[2,2]=1

for(i in 2:nom){

ia = i+1

ib = ia - 1

ic = ia - 2

c = 2*(i-1) + 1

f = i - 1

c = c/i

f = f/i

for(j in 1:ia){

if(j == 1){ z = 0 }

else {z = phi[ib,j-1]}

phi[ia,j] = c*z - f*phi[ic,j]

}

}

for( m in 1:no){

f = sqrt((2*(m-1)+1)/2)

phi[m, ] = phi[m, ]*f

}

return(phi[1:no,1:no])

}

LAMg=LPOLY(6)

ti=c(-1028.89:1868.834) #Environmental gradient

tmin=min(ti)

tmax=max(ti)

# STANDARDIZATION OF AGES FOR THE GRADE 5 POLYNOMY (MODEL CHOSEN BY PAL)

qi= (2*(ti-tmin)/(tmax-tmin))-1

amb_desf=round(-(1+2*(tmin))/2)

# GETTING MATRIX M

x=qi

x0=x*0 + 1

x2=x*x

x3=x2*x

x4=x3*x

x5=x4*x

Mg=cbind(x0,x,x2,x3,x4,x5)

PHg = Mg %*% t(LAMg)

PHIg=PHg

# genetic value of strains

Coefg=as.matrix(read.table("E:\\regressao\\results\\Coef_g.txt",h=F))

EBV=Coefg%*%t(PHIg)

#write.table(as.matrix(cbind(EBV)),"E:\\regressao\\results\\EBV.txt", row.names = FALSE)

EBV_ide_max=matrix(apply(X=EBV[1:100,],MARGIN=2,FUN=max),1,ncol(EBV))

EBV_ide_min=matrix(apply(X=EBV[1:100,],MARGIN=2,FUN=min),1,ncol(EBV))

#EBV ALL LINES + IDEOTYPES IN ALL ENVIRONMENTS

EBVg=as.matrix(rbind(EBV[1:100,],EBV_ide_max,EBV_ide_min))

#COEFFICIENTS WITH THE INCLUSION OF THE IDEOTYPE

CCf=EBVg%*%PHIg%*%solve(t(PHIg)%*%PHIg)

#EBV GENOTYPES + IDEOTYPES

EBVf=CCf%*%t(PHIg)

IN=4 ##NUMBER OF IDEOTYPES

geral <- scale(rbind(EBV[1:100,],EBV_ide_max), center = FALSE, scale = apply(rbind(EBV[1:100,],EBV_ide_max), 2, sd))

DEgeral <- dist(geral)

DEMgeral <- as.matrix(sqrt( (1/ncol(geral))*((DEgeral)^2) ))

GIDgeral <- DEMgeral[1:nrow(geral)-1, nrow(geral)]

desf <- scale(rbind(EBV[1:100,1:amb_desf],EBV_ide_max[,1:amb_desf]), center = FALSE, scale = apply(rbind(EBV[1:100,1:amb_desf],EBV_ide_max[,1:amb_desf]), 2, sd))

DEdesf <- dist(desf)

DEMdesf <- as.matrix(sqrt( (1/ncol(desf))*((DEdesf)^2) ))

GIDdesf <- DEMdesf[1:nrow(desf)-1, nrow(desf)]

fav <- scale(rbind(EBV[1:100,-c(1:amb_desf)],EBV_ide_max[,-c(1:amb_desf)]), center = FALSE, scale = apply(rbind(EBV[1:100,-c(1:amb_desf)],EBV_ide_max[,-c(1:amb_desf)]), 2, sd))

DEfav <- dist(fav)

DEMfav <- as.matrix(sqrt( (1/ncol(fav))*((DEfav)^2) ))

GIDfav <- DEMfav[1:nrow(fav)-1, nrow(fav)]

minimo <- scale(rbind(EBV[1:100,],EBV_ide_min), center = FALSE, scale = apply(rbind(EBV[1:100,],EBV_ide_min), 2, sd))

DEminimo <- dist(minimo)

DEMminimo <- as.matrix(sqrt( (1/ncol(minimo))*((DEminimo)^2) ))

GIDminimo <- DEMminimo[1:nrow(minimo)-1, nrow(minimo)]

GID <- cbind(GIDgeral,GIDdesf,GIDfav,GIDminimo)

rownames(GID)=c("Capixaba_Precoce","Ouro_Negro","Pérola","BRS_Valente","BRS_Campeiro","BRS_Grafite","BRS_Requinte","BRS_Pontal","BRS_Majestoso","BRS_Supremo","BRSMG_Pioneiro",

"BRS_Esplendor","BRS_Cometa","BRS_Expedito","BRS_Estilo","BRS_Notável","Rio_doce","Rudá","Diamante_Negro","Onix","Aporé","Xamego","BR_6-Barriga_verde","SCS_Guará",

"VP_33","VC15","VP_22","Milionário_1732","Rico_1735","FT_120","FT_bonito","Carioca_1070","Carioca_1030","Moruna","Carioca_80","IAC_Carioca","IAC-Una",

"IAC_-_Carioca_Pyatã","IAC_-_Carioca Akytá","IAC_Votuporanga","IAC-Ybaté","IAC-Apuã","IAC_Alvorada","IAC_Formoso","Rio_Tibagi","IAPAR_8-Rio_Negro","IAPAR_16",

"IAPAR_20","IAPAR_44","IAPAR_31","IAPAR_57","IAPAR_65","IPR_Tangará","IPR_Tuiuiú","BR-_IPA_10","BR-_IPA_11-Brígida","IRAÍ","BR-IPAGRO_1-_Macanudo","Preto_Uberabinha",

"BR-2_Grande_Rio","BR-3_Ipanema","BR_1-_Xodó","Varre-Sai","BRSMG_Madrepérola","BRSMG_Talismã","Rico_23","IPR_139","IPR_Uirapurú","IPR_Gralha","IPR_Eldourado",

"IPR_Graúna","IPR_Tiziu","IPR_Campos_Gerais","IPR_Saracura","IAPAR_81","Pampa","IAC_Tunã","IPR_Andorinha","IPR_Colibri","IAC_Imperador","BRS_Esteio","Meia_Noite",

"Porto_Real","Minuano","IAC-Aruã","BRS_Agreste","IAC_Ayso","Macotaço","Rudá_R","IAC-_Diplomata","BRS_Horizonte","IAC-Maravilha","BRS_Ametista","IAPAR_80","Carioca_MG",

"Princesa","IPR_Siriri","IPR_Chopim","RP1","VC_17")

Cultivars=rownames(GID)

grupo=c("P","P","C","P","P","P","C","C","C","P","C","P","C","P","C","C","C","C",

"P","P","C","P","P","C","P","C","P","P","P","P","C","C","C","P","C","C","P","C",

"C","C","C","C","C","C","P","P","C","P","P","C","C","P","C","P","P","C","P","P",

"P","P","P","P","P","C","C","P","C","P","P","C","P","P","C","C","C","P","P","C",

"C","C","P","P","C","P","C","P","C","P","C","P","C","P","C","C","C","C","C","P",

"C","C")

spatial.prob.classificacao <- (1/GID)/(replicate(IN, c(as.numeric(apply((1/GID),1,sum)))))

spatial.prob.recomendacao <- (1/GID)/t((replicate(nrow(EBV[1:100,]), c(as.numeric(apply((1/t(GID)),1,sum))))))

ideotype.rank.classificacao <- lapply(1:IN, function(i){sort(spatial.prob.classificacao[,i],decreasing = TRUE)})

names(ideotype.rank.classificacao) <- paste("ID",1:IN,sep="")

#ideotype.rank

#data.frame(cbind(Cultivars,grupo,ideotype.rank$ID1))

#data.frame(cbind(Cultivars,grupo,ideotype.rank$ID2))

#data.frame(cbind(Cultivars,grupo,ideotype.rank$ID3))

#data.frame(cbind(Cultivars,grupo,ideotype.rank$ID4))

## Recomendation

output.recomendacao<-NULL

for(i in 1:nrow(spatial.prob.recomendacao)){

output.recomendacao[i] <- names(which.max(spatial.prob.recomendacao[i,]))

}

rec <-data.frame(Cultivars=Cultivars,Grain_type=grupo,spatial.prob.recomendacao)

rec

subset(rec, rec$Grain_type=="C")

subset(rec, rec$Grain_type=="P")
