# Supplementary material for: Adaptability and stability analyses of plants using random regression models
Source: PLoS One. 2020 Dec 2;15(12):e0233200. doi: 10.1371/journal.pone.0233200 (PMC7710123; doi:10.1371/journal.pone.0233200)
Supplement: S1 DOI — (DOCX) [file pone.0233200.s007.docx]

**S1 DOI: Dataset.** The dataset are available in the Figshare online repository, at the link: https://doi.org/10.6084/m9.figshare.12668390.v1
