# Supplementary material for: Adaptability and stability analyses of plants using random regression models
Source: PLoS One. 2020 Dec 2;15(12):e0233200. doi: 10.1371/journal.pone.0233200 (PMC7710123; doi:10.1371/journal.pone.0233200)
Supplement: Attachment — Submitted filename: Response to Reviewers.docx [file pone.0233200.s008.docx]

**Response to Reviewers**

The authors of this paper would like to thank the reviewers' comments and suggestions. His excellent tips contributed greatly to the improvement of this work, leading to greater understanding and ease of interpretation. We carefully analyze each comment and seek to meet the requests of both reviewers. Below is a detailed description of each suggestion and the change made to the article, in order to simultaneously serve the reviewers.

**Reviewer #1)**

- The author may hypothesize why using Environmental gradient applying Finlay and Wilkinson is advantageous than environment traits to obtain the Environmental gradient;

Authors reply: We accept the reviewer's suggestion and try to explain this issue better in the text, in the Material e Methods.

- The authors did not compare the proposed methodology to established methodologies. This could enhance the discussion;

Authors reply: We only cited the traditional methodologies, but we did not make comparisons between them, since it was not our goal to compare methodologies: In addition, problems could occur during the analysis, due to our experiments being unbalanced.

- Is it possible to use covariance matrix for the genotypic effect? It was used in the Phaseolus vulgaris L application?

Authors reply: It is possible to incorporate the matrix of genotypic effects in the model used; however, as we did not have this information, we did not use it. We try to show the advantage of using this information in the discussion.

- Line 35-36: Check the reference

Authors reply: we accept this suggestion and modify this reference, updating it.

- Line 47-54: Need reference for theses affirmations

Authors reply: we accept this suggestion and prefer to remove this paragraph.

- Line 66: imbalances or unbalanced? check in the text

Authors reply: we accept this suggestion and change the word in the text to ‘unbalanced’.

- Line 240: humidity to moisture

Authors reply: we accept this suggestion and change in the text.

- Line 267: witnesses to check

Authors reply: we accepted thus suggestion and changed in the text.

Discussion

- Usually, in animals and plants, Legendre polynomials and other random regression are used to model the growth of the individual. In the presented methodology, it was not clear the “biological” meaning of modeling reaction norm using only environmental gradient with only the same priori information. see DOI 10.1007/s00122-013-2243-1. How the model deal with genotype x environment interaction? Is it feasible to extrapolate the presented methodology to unobserved environments? Since the Mixed model approach is used.

Authors reply: As mentioned by the reviewer, random regression models were proposed to model the behavior of genotypes in animal breeding, through longitudinal measurements in space, to model, for example, lactation curves in cattle. Our attempt to apply the RRM in plant breeding is to try to model the behavior of bean cultivars according to a variation in a series of environments, that is, a spatial variation of the genotypes. The reaction norm is a model that captures the variation of genotypes across environments and adapts to each of them a regression to their behavior, in order to assess their adaptability. In addition, as it is a methodology based on the REML/BLUP procedure, it is possible to predict the behavior of a genotype in an environment in which it has not been evaluated, through the behavior of the other genotypes in that environment. However, the smaller the number of environments in which the genotype was evaluated, the lower the accuracy of prediction.

- Line 424-425: How the presented methodology could incorporate the genomic matrix and environmental data in order to increase predictive ability?

Authors reply: In our work, we did not include genomic and environmental information in our model because we did not have this information. Until these experiments were carried out, our program did not have sufficient resources to obtain genomic and environmental data. However, the methodology allows the inclusion of individuals' genomic information, kinship information and environmental data. Such data can be included in the matrices of genotypic effect (pedigree and genomic data) and residual effect (environmental data such as temperature and radiation and others trials information, such as plot positioning via spatial analysis). We added in the ‘Discussion’ section some relevant points in this regard. Line 429-430: Which studies are these? What are the connections between the presented methodology and them?

Authors reply: We understood this doubt and modified this sentence in the text. Now it is better explained in the Introduction.

- Line 431-437: “The fact that an individuals' behavior is not predetermined, is an advantage of the proposed methodology in relation to the traditional methods of analysis of adaptability and stability.” I do not understand this affirmation. The model doesn’t use the environmental gradient defined a priori from observed genotypes?

Authors reply: in this statement, we mean that in the more traditional models of regression analysis the behavior of genotypes over a set of trials is pre-established. For example, Ebehart and Russell propose to adjust a linear regression model as a function of the environmental gradient, which assumes that every genotype has a linear behavior across the environments. If a particular genotype behaves differently from the linear, it will have a bad fit and this will be interpreted as low predictability or low stability. It is in this sense that the use of reaction norms allows to better capture the real behavior of each genotype throughout the environments, not assuming that the response of the genotypes according to the environmental gradient is linear.

- Line 446-454: Advantage over which other methodology? The presented work did not compare with the most common methodologies applied.

Authors reply: Actually, we mean that the use of reaction norms is an advantage over traditional methodologies of adaptability and stability.

- Line 452-454: It was not clear if it was used a cross-validation scenario to obtain these predictive accuracies. If it wasn’t, interpretation regarding predictive accuracy must be pondered.

Authors reply: we understand your question. We do not use a cross-validation scenario because the accuracy is estimated directly through the Predicted Error Variance (PEV). The PEV, in turn, is obtained from the diagonal elements of the matrix of the estimated coefficients for each genotype in the study. Thus, it is not necessary to use a cross-validation scenario to estimate the prediction accuracy.

- Line 460-461: Comparison among mixed models and others approach must be careful since the present work did not evaluate any other approach.

Authors reply: we agree with this suggestion and we think it is better remove this sentence of the text.

- Line 482-524: These paragraphs focus mainly on bean recommendation. Although very important, these do not seem to be the main focus of the study.

Authors reply: We agree with this suggestion and remove these paragraphs from the text. Really, we are not focusing on the recommendation of beans, but on the application of RRM in plant breeding.

Reference

- Many references are in Portuguese or are from books. These type of references should be avoided in order to ensure access to the readers.

Authors reply: As suggested by the reviewer, some of these references have been removed or updated.

**Reviewer #2:**

- The authors argued as if they developed RRM to investigate phenotypic plasticity or reaction norm. For instance, this is reflected in the title, "We propose a new methodology" in the Abstract, and "Thus, the objectives of this investigation were to propose a new methodology for ..." However, this is incorrect as the use of RRM for phenotypic plasticity analysis has been an active research area in animal breeding and ecology for more than decades. You are applying the previously developed methodology to study phenotypic plasticity in plants by treating multi-environment trials as environmental gradients. This manuscript would be much more appealing to plant breeders if written in the context of those works by others.

Authors reply: Thank you for the excellent review. Actually, we are only applying the RRM theory in plant breeding, more explicitly in the performance of genotypes over a set of environments. Thus, we changed the title and objectives, as suggested by the reviewer.

- My second reservation pertains to the Introduction section that lacks a good structure. First, start by framing why adaptability/stability analysis is an important research area in the context of multi-environment trials (MET) and genotype by environment (GEI). Note that MET and GEI were never mentioned in the Introduction in the current version. The term MET first appears in the Methods section and GEI is only referred to in the Discussion section. Second, briefly review rich literature on how and why RRM has been widely used to study plasticity or GEI in animal breeding and ecology. Lastly, clearly state the objective of this paper, which is the application of RRM to investigate reaction norms in plant breeding. Also, just saying linear mixed models coupled with Legendre polynomials is not sufficient. Explicitly mention that the model you are using is RRM. I suggest entirely rewriting the Introduction.

Authors reply: We fully understand the comments made. Thus, we rewrote the Introduction, seeking a greater theoretical basis in scientific literature in order to better justify the work.

- The third major concern is the lack of methodological clarity and smoothness. The current Methods section is written like a step by step software manual with a disconnection between them. Not quite sure how Ij defined in step 1 is relevant to the subsequent steps. Steps in L131 and L144 can be combined with others because they are well-known statistics. I believe the methodology part can be significantly improved.

Authors reply: we understand the comments. Since we are no longer using the work as the proposal for a new methodology, we changed the material and methods, removing the description 'step to step’. The material and methods are now described continuously.

- The utility of RRM is limited in this study because the authors did not use any genetic data (pedigree or genomics). Consider adding one or two paragraphs, regarding the impact of genetics on adaptability and stability analyses in the Discussion section.

Authors reply: We do not use genomic and environmental data in our work because we do not have this information. However, we add in the discussion section how genomic and environmental data can be included in works of this type, as well as what advantages are obtained with the use of this information.

Below are my specific comments.

- Data availability statement. It is a bit strange to say that the data will be available upon acceptance of this manuscript. The data should be made available for reviewers and editors through the reviewing process if you intend to make them open.

Authors reply: we apologize for not providing this information previously. We commit this failure. Now, the dataset is available.

- L47: It was not clear to me what you are referring to by current technologies and lower level technologies.

Authors reply: We removed this paragraph from the text, as it also received criticism from the other reviewer. With the new introduction, we think it is no longer needed.

- L123: How is the vector of phenotypes constructed? Is it ordered by genotypes or trials? This will determine how you specify the variance-covariance terms for g and e.

Authors reply: the vector of phenotypes was first ordered by trials according to environmental gradient and within each trial, ordered by genotypes. The ASReml procedure requires that data be organized in this way for a multivariate model.

- There is an inconsistency in your notation because Kg is on the left side of the Kronecker product, while Sigma_e is on the right side of the Kronecker product.

Authors reply: we made this mistake in the description of the method. Now, we modified it in the text.

- L155, L163, L168: It is misleading to say "at the original scale". You are just predicting breeding values from estimated random regression coefficients.

Authors reply: we accept this suggestion and prefer remove this sentence to avoid misunderstanding.

- L157: What BLUPs are you referring to? I do not see any connection between this and the preceding subsections.

Authors reply: we accept this doubt. Therefore, we prefer remove the term BLUP and to use only the term “genetic value”.

- L173: Clarify how PEV was computed from a random regression model.

Authors reply: we understand this doubt and explain it better in the text, in the Material e Methods. The PEV is obtained from the diagonal elements of the matrix of the estimated coefficients for genotype *i* in trial *j*

- Equation 9: A reference is needed.

Authors reply: We added one reference in the text.

- L204: I did not understand this paragraph. What does it mean by "through the invariance in multi environment trials (MET)"?

Authors reply: we understand this doubt and change in the text, trying to explain better. We wanted to say that the stability can be highlighted as variation regarding the behavior of the ideotype along the MET.

- Genetic material (L216) and Trials (L223) should be placed before the Methods description (L83) according to the flow.

Authors reply: we agree with this suggestion and made the change in the text.

- L218: How population structure of Carioca grains and Black grains were accounted for. I believe this will impact the interpretation of results.

We do not consider the structure of the type of grain, separating the Black and Carioca type, since all genotypes were evaluated together in all environments. In addition, all genotypes were randomized throughout MET. In addition, the set of genotypes presents other sources of variation, such as different cycle durations and different growth habits. Certainly, considering all these variations may change the result, but as the environmental index of Finlay and Wilkinson represents a quality of the environment, it is better estimated when all the values of the trial are used simultaneously.

- L322: It is not evident in Figure 1, which ones are environmental gradients six and eight.

Authors reply: we understand this question and change this sentence in the text.

- L324: This is essential information. I suggest you show a table or a figure related to variance components.

Authors reply: we chose to remove this explanation from the text, as the data needed to justify it was not so clear.

- Figure 1: Clarify why the x-axis is ranging from -1 to 1. This does not agree with Table 1.

Authors reply: we accept this suggestion. In fact, the values between -1 and 1 are a standardization of environmental index. Therefore, we added this description in the Material and Methods, explaining why it is important to standardize the data and how to do it.

- L340: You previously mentioned in L264 that the cultivars of minimal adaptability will not be considered for recommendations. Why do the cultivars of minimal adaptability appear here again?

Authors reply: we made this mistake in the description of the method. It really does not make sense to recommend cultivars of minimal adaptability. Thus, we removed from the Material and Methods and the Results the parts that related to cultivars of minimal adaptability

- L406: Introduce the concept of genotype by environment interactions first in the Introduction section rather than in the Discussion.

Authors reply: we fully agree with this suggestion. Thus, we explain these terms in the Introduction.

- L426: This paragraph should be placed in the Introduction section.

Authors reply: we fully agree with this suggestion and move this paragraph to introduction.

- L446: You did not propose the method (reaction norm analysis using RRM), but you applied.

Authors reply: It was a great suggestion. Thus, we change all this work for the application of RRM and not propose a new method.

Minor comments.

- L117: Consider replacing order of adjustment with order of polynomials

Authors reply: we modify in the text.

- L122, L293, L308: residue -> residual or residuals

Authors reply: we modify in the text.

- L200: Why na appears twice for k = 1 and k = 4?

Authors reply: We made a mistake during the writing. Now we modify in the text.

- L281: table 1 -> Table 1.

Authors reply: we modify in the text.

- L293: Consider replacing grade six with order six. Also, see L306.

Authors reply: we agree and modify in the text.

- Table 2: The column LOG L is redundant if all models converged.

Authors reply: we accept this suggestion and remove this column as well as make other changes.

- L313: There is no need to redefine DEG, AIC, BIC, PAL, LRT, H, and D again.

Authors reply: perfect suggestion. We modify in the text.

- L322: figure 1 -> Figure 1

Authors reply: we modify in the text.

- L401-402: Be consistent with the use of trials vs. environments

Authors reply: we understand that comment and try to be more careful.
