# Supplementary material for: Adaptability and stability analyses of plants using random regression models
Source: PLoS One. 2020 Dec 2;15(12):e0233200. doi: 10.1371/journal.pone.0233200 (PMC7710123; doi:10.1371/journal.pone.0233200)
Supplement: Attachment — Submitted filename: Response to Reviewers.docx [file pone.0233200.s009.docx]

**Response to Reviewers**

Dear reviewers,

The authors of this paper would like to thank the reviewers' comments and suggestions. We believe that this tips contributed greatly to the improvement of this work, leading to greater understanding and ease of interpretation. We carefully analyze each comment and seek to meet the requests of both reviewers. Below is a detailed description of each suggestion and the change made to the article, in order to simultaneously serve the reviewers.

**Reviewer #1)**

- L.48: Replace “imbalance” to “unbalanced”
- L.155: Replace “Genetic imbalance” to “Genetic Unbalanced”
- L. 293: Replace "Witnesses" to "check"
- L. 294: Replace "as witnesses to check" "as check"
- L.248: Equation 9. Reference needed
- L.381: Replace "Witnesses" to "check"
- L. 657: Remove

Authors reply: We would like to thank you the reviewer for the suggestions. We think they made this work better. We have been made the changes in the text.

**Reviewer #2:**

- L103: This is incorrect. Henderson proposed BLUP, linear mixed model, and mixed model equations, but not RRM. The initial form of RRM was first proposed by Kirkpatrick et al. 1990 [1] and later extended by Schaeffer and Dekkers 1994 [2] and Meyer and Hill 1997 [3].

[1] Kirkpatrick et al. 1990 Analysis of the inheritance, selection and evolution of growth trajectories. Genetics 124: 979–993.

[2] Schaeffer LR, Dekkers JCM. Random regressions in animal models for test-day production in dairy cattle. Proc 5th World Congress on Genetics Applied to Livestock Production; 1994; Guelph, 18:443-446.

[3] Meyer K, Hill WG. Estimation of genetic and phenotypic covariance functions for longitudinal or repeated records by restricted maximum likelihood. Livest Prod Sci. 1997; 47:185–200.

Authors reply: We apologize for the mistake. In this way, we understood the suggestions pointed out by the reviewer and made changes to the text, as suggested. We would like to thank you for the suggestions.

- It is important to note that the current work is not the first time to apply RRM to univariate or multivariate plant breeding data. The authors failed to refer to the earlier work of Sun et al. 2017 [4], Ly et al. 2018 [5], Momen et al. 2019 [6], and Baba et al. 2020 [7] in the Introduction section. Put them after animal breeding literature you referred and mention their contribution/relevance with respect to the current work.

[4] Sun et al. Multitrait, random regression, or simple repeatability model in high-throughput phenotyping data improve genomic prediction for wheat grain yield. Plant Genome. 2017; 10. pmid:28724067

[5] Ly et al. Whole-genome prediction of reaction norms to environmental stress in bread wheat (Triticum aestivum 736 L.) by genomic random regression. F Crop Res. 2018;216.

[6] Momen et al. Predicting longitudinal traits derived from high-throughput phenomics in contrasting environments using genomic Legendre polynomials and B-splines. G3: Genes, Genomes, Genetics. 2019.  9:3369-3380.

[7] Baba et al. Multi-trait random regression models increase genomic prediction accuracy for a temporal physiological trait derived from high-throughput phenotyping. PLoS ONE 2020. 15(2): e0228118.

In sum, the introduction section should be framed in the context of earlier work by others.

Authors reply: Again, we made a mistake in not mentioning some works that used RRM previously. Then, we read these articles recommended by the reviewer and made the corrections in the text. Thank you for the suggestion.

- L185: I believe you meant the lowest and highest averages rather than lower and higher averages.

Authors reply: Yes, we wanted to mean the lowest and highest averages. Thank you for the suggestion. We changed these words in the text.

- L191-194: What do you mean by "adjusted"? Perhaps "adjusted" is not the best term to use here.

Authors reply: It is true; the term adjusted was not the best in this sentence. We changed the word in the text to ‘fitted’.

- L196, Equation 3: Why don't you include fixed random regression coefficients designed to capture the mean trajectory of environmental gradients? Almost all of the previous RRM literature include this term to account for the mean trend.
- Thank for you doubt, reviewer. The reason why we did not use the coefficients for the fixed part of the model was that, specifically in this work, we were not interested in estimating a mean for a not existing environment throughout the MET. However, as the reviewer said that this is common in the literature, we also affirm that this could have been done, without problems for the model. It was just our option to choose this model, with the fixed part without the drawing of a trajectory.
- L248: Provide a reference for equation 9.

Authors reply: We added the reference in the text for this equation.

- L264: Remove "thus"

Authors reply: the word “thus” was removed from the text.

- L293, L375: What witnesses are? I am not familiar with this term. I believe you meant checks?
- Authors reply: we accepted this suggestion and changed the word in the text to ‘checks’.
- Table 2: Clarify what H or D means (first column) in the table caption.

Authors reply: We put the D and H means in the table 2 caption.

- L765: Smith et al. 2015 has been cited twice. Ref 1 and Ref 73.

Authors reply: We fixed those citations in the text.

- Ref 20. Wrong journal title. Should be Journal of Dairy Science.

Authors reply: We fixed this reference in the text.

- Ref 38: It says "Available: file:///C:/Users/Sandrinho/Downloads/artículo_redalyc_253021631009.pdf". Note that this is the author's locale file on a computer. Readers will not have access to it.
- Authors reply: We fixed this reference in the text.
- Lastly, I agree with reviewer 2 that references are used to support or defend your claims you made in your research. Note that PLOS ONE has a wide range of readers from around the world. I could not assess many references such as 9, 38-39, 42, 48-52, 61, 72, 76 because they are not written in English. Consider replacing them, if possible, with more suitable references that everyone can read, understand, and discuss.
- Authors reply: thank you for the comment. We tried to change these references written in Portuguese and we did this for most of them. We just could not find works corresponding to references 38, 39 and 52, since they are very specific references to VCU trials and common beans trials in Brazil, with no similar articles in international journals.

These references are in the following order in the text:

[41] Resende MDV de, Duarte JB. Precisão e controle de qualidade em experimentos de avaliação de cultivares. Pesqui Agropecuária Trop. 2007;37: 182–194.

[42] Melo CLP de, Carneiro, José Eustáquio de Souza Carneiro PCS, Cruz CD, Barros, Everaldo Gonçalves de Moreira MA. Linhagens de feijão do cruzamento “Ouro Negro” x “Pérola” com características agronômicas favoráveis. Pesqui Agropecuária Bras. 2006;41: 1593–1598.

[54] Ramalho MAP, Abreu A de FB, Santos PSJ dos. Interações genótipos x épocas de semeadura, anos e locais na avaliação de cultivares de feijão nas regiões Sul e Alto Paranaíba em Minas Gerais. Ciência e Agrotecnologia. 1998;22: 175–181.
