# Supplementary material for: Adaptability and stability analyses of plants using random regression models
Source: PLoS One. 2020 Dec 2;15(12):e0233200. doi: 10.1371/journal.pone.0233200 (PMC7710123; doi:10.1371/journal.pone.0233200)
Supplement: Attachment — Submitted filename: Response to Reviewers.docx [file pone.0233200.s010.docx]

**Response to Reviewers**

Dear editor and reviewers,

The authors of this paper would like to thank the reviewers' comments and suggestions. We believe that this tips contributed greatly to the improvement of this work, leading to greater understanding and ease of interpretation. We carefully analyze each comment and seek to meet the requests of both reviewers. Below is a detailed description of each suggestion and the change made to the article, in order to simultaneously serve the editor and the reviewers.

**Editor:**

- Throughout the text use "common bean" instead of just "bean";

Authors reply: Thank you for the suggestion. We made the changes in the text.

- quote all R packages used for the analyzes;

Authors reply: Thank you for the suggestion. We added the packages on the article and on the supporting information.

- In the Discussion, insert a final paragraph about what advances the proposed method has in relation to the dozens of methods in the literature;

Authors reply: Thanks for the suggestion. We added a paragraph at the end of the Discussion showing the advantages of the proposed method. In addition, over the Introduction and Discussion sections, we show a more detailed description of the method's advantages over those already available in the literature.

- Add in the conclusions the best genotypes identified by RR models.

Authors reply: Thank you for the suggestion. We did that.

**Reviewer #1:**

Please, check if the term "imbalances" is correct

Authors reply: Thank you for the suggestion. We check that in the text and made some changes aiming a better understanding.

I did not understand equation 9 and I did not find a direct connection between the citation. Considering that part of your conclusions is based on it, I recommend that you explain it better or reference it.

Authors reply: Dear reviewer, thanks for the suggestion. We try to understand your doubts about the accuracy equation. We used the equation adapted from Gilmour et al. (1990), using the *PEV* (Predictor Error Variance) and *Kg* (covariance coefficients for genotypic effect) matrices. However, using Legendre orthogonal polynomials, we made an adaptation in the equation, pre and post multiplying the *PEV* and *Kg* by the *Ф_ijm_* matrix (Legendre's *m-th* polynomial for the *j-th* trial and the *i-th* genotype), in order to transform the values obtained from the Legendre scale to the original scale, as proposed by Kirkpatrick et al. 1990. In addiction, we add the two references cited below in the article.

[1] Gilmour, A.R., Gogel, B.J., Cullis, B.R., and Thompson, R. ASReml User Guide Release 3.0 VSN International Ltd, Hemel Hempstead, HP1 1ES, UK; 2009. [www.vsni.co.uk](http://www.vsni.co.uk)

[2] Kirkpatrick M, Lofsvold D, Bulmer M. Analysis of the inheritance, selection and evolution of growth trajectories. Genetics. 1990;124: 979–993.
